# Neutrophil–lymphocyte ratio as a potential marker for differential diagnosis between spinal tuberculosis and pyogenic spinal infection

**DOI:** 10.1186/s13018-022-03250-x

**Published:** 2022-07-21

**Authors:** Hao Liu, Yin Li, Jiang Yi, Wei Zhou, Shujie Zhao, Guoyong Yin

**Affiliations:** Department of Orthopedics, the First Affiliated Hospital of Nanjing Medical University, Nanjing, 210029 Jiangsu China

**Keywords:** Neutrophil–lymphocyte ratio, Spine tuberculosis, Pyogenic spinal infection, Differential diagnostic value

## Abstract

**Objective:**

Distinguishing spinal tuberculosis and pyogenic spinal infection is extremely important. The neutrophil–lymphocyte ratio (NLR), a simple indicator, has been shown to be a novel inflammatory marker. The objective of our study was to determine whether the NLR could be a potential indicator for discriminating spinal tuberculosis (STB) from pyogenic spinal infection (PSI).

**Methods:**

We compared the clinical and laboratory characteristics of 146 patients diagnosed with STB and 60 participants with PSI from the First Affiliated Hospital of Nanjing Medical University. The NLR's diagnostic ability for differential diagnosis was assessed and compared to other hematological indicators, including the platelet–lymphocyte ratio (PLR).

**Results:**

The NLR in STB patients was considerably lower than that in PSI patients [3.85 (2.70–5.71) vs. 10.82 (6.79–17.62), *P* < 0.001]. An NLR of 6.742 was proposed as an optimal cutoff value for distinguishing patients with STB from those with PSI (sensitivity 78.33%, specificity 83.56%). However, the NLR's area under the curve [0.87, 95% confidence interval (CI) 0.81–0.92] was considerably higher than that of the PLR (0.73, 95% CI 0.65–0.80; *P* < 0.0001).

**Conclusion:**

NLR levels could be a valuable laboratory diagnostic for distinguishing patients with STB from those who have PSI.

## Introduction

Tuberculosis (TB) is a leading cause of infectious disease death in adults globally, with almost 30,000 new cases diagnosed each day [1]. Spinal tuberculosis (STB) accounts for more than half of skeletal TB cases, while skeletal TB accounts for more than 10% of extrapulmonary TB cases [2]. Throughout history, TB has been considered a disease of the poor, and it indirectly causes economic burden among societies [3]. In developing nations, STB is more common, and one of its features is delayed diagnosis [4]. Approximately 10–40% percent of patients with STB develop neurological impairment, which leads to tetraplegia in some severe cases [5]. As a result, it is important to diagnose and treat STB as early as possible. The diagnosis of STB is challenging largely due to the similarity of its clinical and imaging findings to those of pyogenic spinal infection (PSI); therefore, it is essential to identify additional biomarkers for the differential diagnosis [6].

The neutrophil-to-lymphocyte ratio (NLR) has recently acquired prominence as a simple and low-cost biomarker of systemic inflammation [7]. Because the NLR includes neutrophils and lymphocytes in its computation, it is thought to be more reliable than other absolute counts [8, 9]. The prognostic or diagnostic value of the NLR or the platelet‐to‐lymphocyte ratio (PLR), which is another commonly used marker, has been evaluated in cardiovascular diseases, inflammatory diseases, coronavirus disease 2019, rheumatoid arthritis, prostate cancer, and several types of cancer [7, 8, 10–14]. Previous research has demonstrated that the NLR can be used to distinguish patients with pulmonary tuberculosis from those with bacterial community-acquired pneumonia [15, 16], and the PLR was considered to be a marker for identifying tuberculosis infection in COPD patients [17].

However, to our knowledge, no research has been conducted on the role of the NLR in the differential diagnosis of STB and PSI. Based on this, the main purpose of this study was to investigate the role of the NLR in discriminating patients with STB from those with PSI and to compare the NLR's diagnostic abilities to those of the PLR.

## Materials and methods

### Patients

Our study is a retrospective analysis. All patient-related data were obtained from the First Affiliated Hospital of Nanjing Medical University from January 2007 to December 2021. We examined the demographic characteristics, clinical information, and laboratory data of 146 STB patients and 60 PSI patients. This study was favorably approved by the Ethical Committee of the First Affiliated Hospital of Nanjing Medical University (Ethical Committee Number: 2021-SR-521).

Exclusion criteria included antibiotic treatments for more than 24 h at the time of enrollment, conditions known to affect total and differential WBC counts such as hematologic disorders, chronic inflammatory conditions, under treatment with steroids, and/or history of steroid use within 3 months before admission, history of chemotherapy or radiotherapy within 4 weeks before enrollment, absence of related WBC differential count data.

### Diagnosis of STB or PSI

The patients were all clinically diagnosed and confirmed to have STB or PSI (vertebral osteomyelitis, pyogenic spondylitis, spondylodiscitis, surgical site infection following spine surgery, epidural abscess) on the basis of clinical, laboratory, radiologic evaluations and clinical response to anti-TB drugs or antimicrobial therapy.

### Hematological indices

The neutrophil, lymphocyte, monocyte, and platelet counts were obtained from medical records. The NLR and PLR were calculated as follows: NLR = neutrophil count/lymphocyte count and PLR = platelet count/lymphocyte count. All of the above variables were compared between STB and PSI patients.

### Statistical analysis

Normally distributed parameter data were analyzed by Student's t test, and for nonnormally distributed data, we used the Mann–Whitney U test. Depending on the data distribution, continuous variables were reported as the mean ± standard deviation or median (25–75% interquartile range). Numbers (*n*) and percentages (%) were used to express categorical variables. The Chi-square test was used to examine qualitative variables. Receiver operating characteristic (ROC) curves were used to summarize specificity and sensitivity. The most relevant cutoff values for the NLR, PLR, and other parameters were determined using the maximum Youden index. *P* values of 0.05 or less were considered significant, and 95% confidence intervals (CIs) were calculated. SPSS 26.0, GraphPad Prism 9.0.0, and MedCalc 19.6.4 were used to conduct all statistical analyses.

## Results

### Clinical data and laboratory tests

**Fig. 1:**
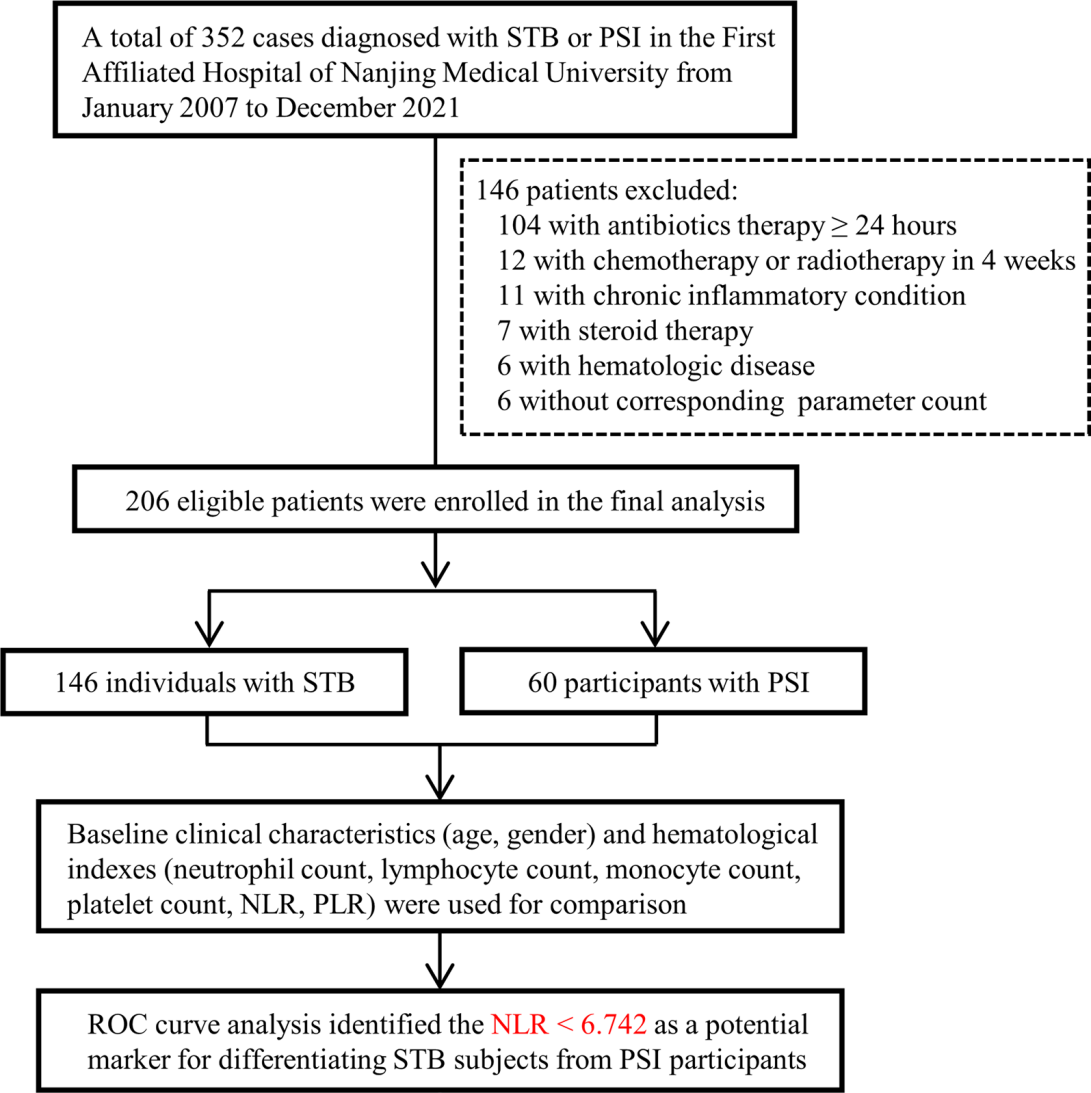
Flowchart of patient inclusion and analysis procedures. *STB* spinal tuberculosis, *PSI* pyogenic spinal infection, *ROC* receiver operating characteristic, *NLR* neutrophil-to-lymphocyte ratio

A total of 206 patients were included in this study (Fig. 1), of which 146 patients were diagnosed with STB and 60 had PSI. Patients with STB and PSI had an average age of 55.70 ± 17.16 and 63.68 ± 11.52 years, respectively. A total of 53.42% (78/146) of STB patients were male, and 56.67% (34/60) of PSI patients were male. Gender differences between STB and PSI individuals were not observed (*P* = 0.671). Table 1 shows the demographics and baseline clinical features of the two groups. The median NLR values were 3.85 (STB group) and 10.82 (SI group). Patients with STB had considerably lower total neutrophil counts, monocyte counts, NLRs, and PLRs than patients with PSI, although the lymphocyte counts and platelet counts were significantly higher in STB patients than in PSI patients (Table 1).

**Table 1:** Baseline clinical characteristics of the population

Parameters	STB	PSI	*P*
Age, years (mean ± SD)	55.70 ± 17.16	63.68 ± 11.52	= 0.001
Gender (male/female)	78:68	34:26	= 0.671
Neutrophil (× 10^9^/L)	6.23 (5.46–7.48)	9.12 (7.52–10.95)	< 0.001
Lymphocyte (× 10^9^/L)	1.60 (1.19–2.01)	0.88 (0.58–1.17)	< 0.001
Monocyte (× 10^9^/L)	0.43 (0.36–0.57)	0.54 (0.39–0.63)	= 0.029
Platelet (× 10^9^/L)	310 (271–347)	248 (234–272)	< 0.001
NLR	3.85 (2.70–5.71)	10.82 (6.79–17.62)	< 0.001
PLR	191.9 (150.6–255.8)	292.9 (213.4–399.5)	< 0.001

Values are shown as the mean, standard deviation, median (IQR), or numbers. Differences between groups were analyzed using Student’s t test or the Mann–Whitney U test for continuous variables and the Chi-square test for categorical variables

*STB* spinal tuberculosis, *PSI* pyogenic spinal infection, *NLR* neutrophil–lymphocyte ratio, *PLR* platelet–lymphocyte ratio

### Differential diagnostic value of the NLR in discriminating STB from PSI

**Fig. 2:**
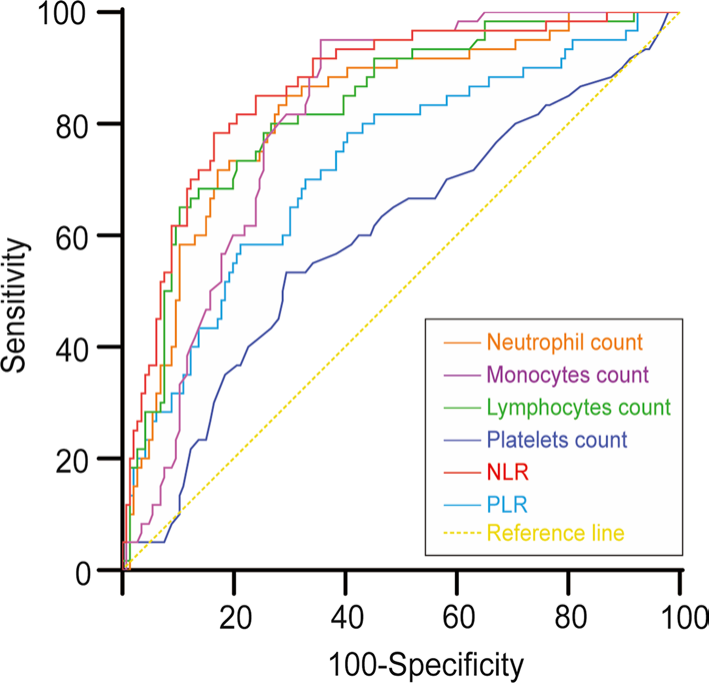
ROC curves were used to assess the diagnostic value of various parameters. *NLR* neutrophil–lymphocyte ratio, *PLR* platelet–lymphocyte ratio

**Fig. 3:**
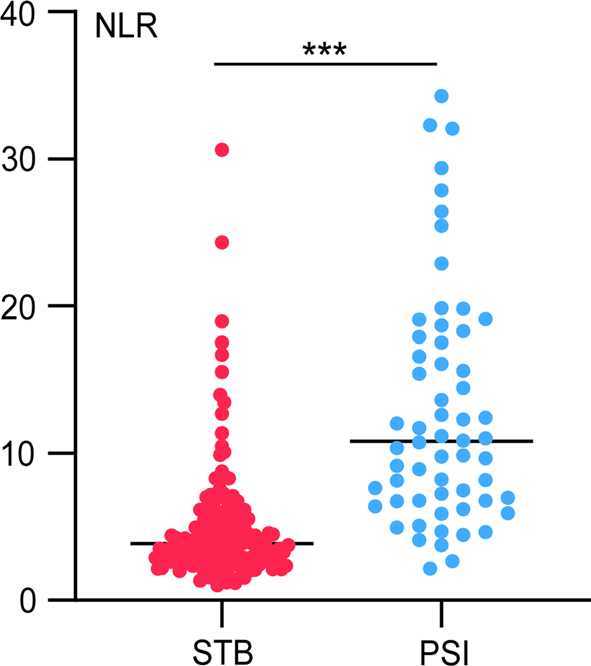
The distribution of NLR of PSI and STB patients. *STB* spinal tuberculosis, *PSI* pyogenic spinal infection, *NLR* neutrophil–lymphocyte ratio

The area under the curve (AUC) and the maximum Youden index were obtained to identify the most useful cutoff levels. The AUC values for the parameters were follows: neutrophil count, 0.82 (95% CI 0.76–0.88); lymphocyte count, 0.83 (95% CI 0.77–0.89); monocyte count, 0.60 (95% CI 0.51–0.68); platelet count, 0.81 (95% CI 0.75–0.87); NLR, 0.87 (95% CI 0.81–0.92); and PLR, 0.73 (95% CI 0.65–0.80) (Table 2 and Fig. 2). Among the variables mentioned above, the NLR has the greatest area under the curve (AUC) value. Furthermore, the cutoff value for the NLR was less than 6.742. The NLR has a sensitivity and specificity of 83.56% and 78.33%, respectively, for distinguishing STB patients from SI participants when using this cutoff value (Table 2). At a cutoff value of 207.7, the sensitivity and specificity of PLR were 78.33% and 59.59%, respectively. The distribution of the NLR of STB patients was lower than that of PSI subjects (^***^P < 0.001) (Fig. 3).

**Table 2:** ROC curves were used to analyze the diagnostic utility of various factors in distinguishing STB from PSI

Markers	AUC	95%CI	Cutoff	Sensitivity (%)	Specificity (%)	Maximum Youden index
Neutrophil count	0.82	0.76–0.88	6.996	85.00	70.55	0.556
Lymphocyte count	0.83	0.77–0.89	0.937	65.00	89.73	0.547
Monocyte count	0.60	0.51–0.68	0.525	53.33	70.55	0.239
Platelet count	0.81	0.75–0.87	292.5	95.00	64.38	0.594
NLR	0.87	0.81–0.92	6.742	78.33	83.56	0.619
PLR	0.73	0.65–0.80	207.7	78.33	59.59	0.379

*AUC* area under the curve, *CI* confidence interval, *NLR* neutrophil–lymphocyte ratio, *PLR* platelet–lymphocyte ratio

## Discussion

Spinal tuberculosis is the most common kind of extrapulmonary tuberculosis [18]. The majority of STB patients have experienced symptoms for several months at the time of diagnosis, and most cases of STB remain undiagnosed [19, 20]. Early clinical manifestations, laboratory findings, and imaging abnormalities lack specificity and may not be distinct enough to distinguish it from its close mimics, particularly pyogenic spinal infection [21]. If the diagnosis can be identified in a timely manner, it will not only help delay the progression of patients but also reduce the economic pressure and avoid the occurrence of spinal deformities [20]. Accordingly, a precise differential diagnosis between STB and PSI is essential in clinical practice.

Various stressful events, particularly systemic inflammation, also lead to elevated neutrophil counts and decreased lymphocyte counts [22]. This suggests one method for NLR to operate as a barometer in a variety of clinical conditions, and the NLR has been considered a diagnostic indicator for acute bacterial meningitis [7], prostate cancer [14], and pulmonary tuberculosis [15, 16]. The NLR and PLR have continued to draw attention as novel markers in many studies [13], as they are both simple, economic, and easily measurable markers that can be directly calculated from routine blood tests at admission [23, 24].

Our results demonstrated differences in those parameters between patients with STB and PSI. The NLR (AUC, 0.87, 95% CI 0.81–0.92) showed the highest diagnostic value in distinguishing STB from PSI. An NLR < 6.742 was suggested as the optimal cutoff value for discriminating patients with STB from patients with PSI; therefore, appropriate diagnosis and intervention may need to be done quickly.

The PLR and other parameters (such as neutrophil or monocyte count) were also analyzed in our study, and they showed lower AUCs than NLR. It should also be mentioned that neutrophil counts showed remarkable discriminative value, but less so than the NLR. Furthermore, the NLR is easily available at most institutions and is more easily affordable to obtain than other markers. Therefore, it is proposed that the NLR might be a valuable marker for differentiating STB from PSI.

There are various limitations to this study that should be highlighted. First, this is a nonrandomized single-center study with a limited sample size. More extensive prospective studies are needed. Second, the cutoff value for the NLR in this study was established mainly from in-hospital individuals in the Chinese population. The optimal cutoff value may need to be assessed outside of China to account for potential differences across ethnicities and to account for different reference ranges for hematological parameters.

In conclusion, our study suggested that the NLR, which can easily be evaluated, may be a good marker for discriminating patients with STB from patients with PSI. In our situation, the NLR's differential diagnostic ability outperformed that of the PLR. The NLR should be included in regular tests in individuals with an uncertain diagnosis between STB and PSI.

## Data Availability

The raw data will be made available from the authors upon reasonable request.

## Abbreviations

NLR: Neutrophil–lymphocyte ratio
STB: Spinal tuberculosis
PSI: Pyogenic spinal infection
PLR: Platelet–lymphocyte ratio
TB: Tuberculosis
ROC: Receiver operating characteristic
CI: Confidence interval
AUC: Area under the curve

## References

[1] Jain AK, Kumar J (2013). Tuberculosis of spine: neurological deficit. Eur Spine J.

[2] Khanna K, Sabharwal S (2019). Spinal tuberculosis: a comprehensive review for the modern spine surgeon. Spine J.

[3] Nathavitharana RR, Friedland JS (2015). A tale of two global emergencies: tuberculosis control efforts can learn from the Ebola outbreak. Eur Respir J.

[4] Merino P, Candel FJ, Gestoso I, Baos E, Picazo J (2012). Microbiological diagnosis of spinal tuberculosis. Int Orthop.

[5] Schirmer P, Renault CA, Holodniy M (2010). Is spinal tuberculosis contagious?. Int J Infect Dis.

[6] Chang M-C, Wu HTH, Lee C-H, Liu C-L, Chen T-H (2006). Tuberculous spondylitis and pyogenic spondylitis: comparative magnetic resonance imaging features. Spine.

[7] Mentis AFA, Kyprianou MA, Xirogianni A, Kesanopoulos K, Tzanakaki G (2016). Neutrophil-to-lymphocyte ratio in the differential diagnosis of acute bacterial meningitis. Eur J Clin Microbiol Infect Dis.

[8] Forget P, Khalifa C, Defour J-P, Latinne D, Van Pel M-C, De Kock M (2017). What is the normal value of the neutrophil-to-lymphocyte ratio?. BMC Res Notes.

[9] Dirican A, Kucukzeybek BB, Alacacioglu A (2015). Do the derived neutrophil to lymphocyte ratio and the neutrophil to lymphocyte ratio predict prognosis in breast cancer?. Int J Clin Oncol.

[10] Karimi A, Shobeiri P, Kulasinghe A, Rezaei N (2021). Novel systemic inflammation markers to predict COVID-19 prognosis. Front Immunol.

[11] Cai J, Li H, Zhang C (2021). The neutrophil-to-lymphocyte ratio determines clinical efficacy of corticosteroid therapy in patients with COVID-19. Cell Metab.

[12] Yang A-P, Liu J-P, Tao W-Q, Li H-M (2020). The diagnostic and predictive role of NLR, d-NLR and PLR in COVID-19 patients. Int Immunopharmacol.

[13] Jin Z, Cai G, Zhang P (2021). The value of the neutrophil-to-lymphocyte ratio and platelet-to-lymphocyte ratio as complementary diagnostic tools in the diagnosis of rheumatoid arthritis: a multicenter retrospective study. J Clin Lab Anal.

[14] Xu Z, Zhang J, Zhong Y (2021). Predictive value of the monocyte-to-lymphocyte ratio in the diagnosis of prostate cancer. Medicine.

[15] Yoon N-B, Son C, Um S-J (2013). Role of the neutrophil-lymphocyte count ratio in the differential diagnosis between pulmonary tuberculosis and bacterial community-acquired pneumonia. Ann Lab Med.

[16] Jeon YL, Lee W-I, Kang SY, Kim MH (2019). Neutrophil-to-monocyte-plus-lymphocyte ratio as a potential marker for discriminating pulmonary tuberculosis from nontuberculosis infectious lung diseases. Lab Med.

[17] Chen G, Wu C, Luo Z, Teng Y, Mao S (2016). Platelet-lymphocyte ratios: a potential marker for pulmonary tuberculosis diagnosis in COPD patients. Int J Chron Obstruct Pulmon Dis.

[18] Pu X, Zhou Q, He Q (2012). A posterior versus anterior surgical approach in combination with debridement, interbody autografting and instrumentation for thoracic and lumbar tuberculosis. Int Orthop.

[19] Mann TN, Davis JH, Walzl G (2021). Candidate biomarkers to distinguish spinal tuberculosis from mechanical back pain in a tuberculosis endemic setting. Front Immunol.

[20] Wang B, Gao W, Hao D (2020). Current study of the detection and treatment targets of spinal tuberculosis. Curr Drug Targets.

[21] Garg RK, Malhotra HS, Kumar N (2019). Spinal tuberculosis: still a great mimic. Neurol India.

[22] Zahorec R (2001). Ratio of neutrophil to lymphocyte counts–rapid and simple parameter of systemic inflammation and stress in critically ill. Bratisl Lek Listy.

[23] Zhao Y, Yue J, Lei P (2021). Neutrophil-lymphocyte ratio as a predictor of delirium in older internal medicine patients: a prospective cohort study. BMC Geriatr.

[24] Liaw F-Y, Huang C-F, Chen W-L (2017). Higher platelet-to-lymphocyte ratio increased the risk of sarcopenia in the community-dwelling older adults. Sci Rep.

